# A Genetic Screen Reveals Arabidopsis Stomatal and/or Apoplastic Defenses against *Pseudomonas syringae* pv. *tomato* DC3000

**DOI:** 10.1371/journal.ppat.1002291

**Published:** 2011-10-06

**Authors:** Weiqing Zeng, Alexandre Brutus, James M. Kremer, John C. Withers, Xiaoli Gao, A. Daniel Jones, Sheng Yang He

**Affiliations:** 1 Department of Energy (DOE)-Plant Research Laboratory, Michigan State University, East Lansing, Michigan, United States of America; 2 Department of Microbiology and Molecular Genetics, Michigan State University, East Lansing, Michigan, United States of America; 3 Department of Biochemistry and Molecular Biology, Michigan State University, East Lansing, Michigan, United States of America; 4 Genetics Graduate Program, Michigan State University, East Lansing, Michigan, United States of America; 5 Department of Chemistry, Michigan State University, East Lansing, Michigan, United States of America; 6 Department of Plant Biology, Michigan State University, East Lansing, Michigan, United States of America; Australian National University, Australia

## Abstract

Bacterial infection of plants often begins with colonization of the plant surface, followed by entry into the plant through wounds and natural openings (such as stomata), multiplication in the intercellular space (apoplast) of the infected tissues, and dissemination of bacteria to other plants. Historically, most studies assess bacterial infection based on final outcomes of disease and/or pathogen growth using whole infected tissues; few studies have genetically distinguished the contribution of different host cell types in response to an infection. The phytotoxin coronatine (COR) is produced by several pathovars of *Pseudomonas syringae*. COR-deficient mutants of *P. s. tomato* (*Pst*) DC3000 are severely compromised in virulence, especially when inoculated onto the plant surface. We report here a genetic screen to identify Arabidopsis mutants that could rescue the virulence of COR-deficient mutant bacteria. Among the *susceptible to coronatine-deficient Pst DC3000* (*scord*) mutants were two that were defective in stomatal closure response, two that were defective in apoplast defense, and four that were defective in both stomatal and apoplast defense. Isolation of these three classes of mutants suggests that stomatal and apoplastic defenses are integrated in plants, but are genetically separable, and that COR is important for *Pst* DC3000 to overcome both stomatal guard cell- and apoplastic mesophyll cell-based defenses. Of the six mutants defective in bacterium-triggered stomatal closure, three are defective in salicylic acid (SA)-induced stomatal closure, but exhibit normal stomatal closure in response to abscisic acid (ABA), and *scord7* is compromised in both SA- and ABA-induced stomatal closure. We have cloned *SCORD3*, which is required for salicylic acid (SA) biosynthesis, and *SCORD5*, which encodes an ATP-binding cassette (ABC) protein, AtGCN20/AtABCF3, predicted to be involved in stress-associated protein translation control. Identification of *SCORD5* begins to implicate an important role of stress-associated protein translation in stomatal guard cell signaling in response to microbe-associated molecular patterns and bacterial infection.

## Author Summary

Pathogen entry into host tissue is a critical first step in causing infection. For foliar bacterial plant pathogens, natural surface openings, such as stomata, are important entry sites into the leaf apoplast (internal intercellular spaces). Recent studies have shown that plants respond to surface-inoculated bacterial pathogens by reducing stomatal aperture as part of the innate immune response to restrict bacterial invasion. Once inside plant tissue, bacteria encounter defenses in the apoplast. To counter host defenses during invasion and in the apoplast, bacterial pathogens produce a variety of virulence factors, such as the polyketide toxin coronatine produced by *Pseudomonas syringae* pv. *tomato* (*Pst*) DC3000. Coronatine-deficient *Pst* DC3000 mutants are compromised in virulence, especially when inoculated onto the plant surface. In this study, we conducted a random genetic screen to identify Arabidopsis mutants that could rescue the virulence of coronatine-deficient mutant bacteria and obtained three classes of Arabidopsis mutants: those that are defective in stomatal closure only, those defective in apoplastic defense only, and those compromised in both stomatal closure and apoplastic defenses. The isolation of these host mutants highlight the important role of COR, a molecular mimic of the plant hormone jasmonate, in overcoming both stomatal and apoplastic defenses during *Pst* DC3000 infection.

## Introduction

*Pseudomonas syringae* pv. *tomato* (*Pst*) strain DC3000 is a Gram-negative bacterium that infects tomato and Arabidopsis and is a model pathogen used to investigate molecular mechanisms underlying plant-pathogen interactions [1]–[3]. In nature, *P. syringae* strains often exhibit an epiphytic phase (i.e., surviving and/or multiplying on the plant surface) before entering the intercellular space of the host through wounds or natural openings such as stomata. Once inside the apoplastic space of a susceptible host plant, *P. syringae* can multiply to high levels within days, a process aided by many virulence factors including secreted phytotoxins and effector proteins delivered into the host cells through the type III secretion system (T3SS). Successful colonization of *P. syringae* in the apoplastic space eventually results in the development of disease symptoms, which usually include localized tissue necrosis and discoloration.

Coronatine (COR) is a non-host-specific phytotoxin produced by several pathovars of *P. syringae*, including *Pst* DC3000 [4]–[8]. COR has been implicated in the inhibition of stomatal closure to facilitate bacterial invasion, promotion of bacterial multiplication and persistence *in planta*, induction of disease symptoms, and enhancement of disease susceptibility in uninfected parts of the plant [7], [9]–[19]. COR shows remarkable structural similarity to the active form of the plant hormone jasmonoyl isoleucine (JA-Ile), and targets the JA receptor complex directly [20]–[22]. Indeed, COR serves as a potent inducer of JA-associated responses and expression of JA-responsive genes in plants [7], [12], [23], [24].

The molecular mechanism(s) by which COR-mediated activation of JA signaling results in various virulence roles of COR throughout the bacterial infection cycle remains to be determined. COR-deficient mutants of *Pst* DC3000 are able to multiply to high levels in salicylic acid (SA)-deficient Arabidopsis plants *nahG* and *sid2*, suggesting that COR may be required to overcome SA-mediated defenses [12], [14], [19]. Indeed, SA-dependent expression of pathogenesis-related (*PR*) genes is suppressed in a COR-dependent manner during *Pst* DC3000 infection in tomato plants [15], [25]. However, such COR-dependent suppression of *PR* gene expression has not yet been observed in *Pst* DC3000 infection of Arabidopsis plants [12]. Interestingly, recent studies show that COR can suppress plant defense responses triggered by microbe-associated molecular patterns (MAMPs). For example, COR inhibits MAMP- and bacterium-triggered stomatal closure, which is dependent on SA signaling [14], [19], and COR and JA suppress MAMP-induced callose deposition in leaf mesophyll cells and root cells [26], [27] in Arabidopsis. Taken together, these results collectively indicate that a major function of COR is to suppress interconnected MAMP- and SA-activated defense responses during bacterial infection.

Thus far, attempts to elucidate the role of COR in virulence have relied heavily on known plant defense signaling mutants. We reasoned that an unbiased genetic screen for plant mutants that affect the function of COR in the context of bacterial infection would be useful. Accordingly, we conducted a genetic screen aimed at isolating Arabidopsis mutants that would rescue the virulence of COR-deficient mutant bacterium *Pst* DC3118. This screen allowed us to identify eight *susceptible to coronatine-deficient Pst DC3000* (*scord*) mutants. Six of them are defective in stomatal closure responses to *Pst* DC3118; the other two have a normal stomatal closure response to bacteria, but show weakened apoplastic resistance to both *Pst* DC3000 and *Pst* DC3118. Further characterization of the six stomatal closure mutants revealed defects in salicylic acid (SA) accumulation, SA-triggered stomatal closure, and/or abscisic acid (ABA)-induced stomatal closure. We cloned *SCORD3*, which is required for SA biosynthesis, and *SCORD5*, which encodes an ABC-transporter-family protein (AtGCN20/AtABCF3), predicted to be involved in stress-associated protein translation control. The identification of *SCORD5* revealed potentially a new layer of regulation of MAMP/bacterium-triggered stomatal closure in Arabidopsis.

## Results

### Experimental setup of the genetic screen

When inoculated onto leaves of wild-type Col-0 plants by dipping at 1×10^8^ CFU/ml, COR-deficient mutants of *Pst* DC3000, such as *Pst* DC3118, do not cause significant disease symptoms or multiply to a high level [14], [19]. *Pst* DC3118 carries a Tn*5* insertion in the *cfa6* gene (Figure S1), which is essential for the biosynthesis of COR [5], [7] (Figure S1). However, *Pst* DC3118 exhibits aggressive growth and causes severe disease symptoms in Arabidopsis mutants that are defective in MAMP signaling, or in biosynthesis or signaling of ABA and SA [12], [14], [19]. For example, the *ost1-2* mutant, defective in a guard cell/ABA signaling kinase, OST1 [28], [29], allows *Pst* DC3118 to multiply to high levels and cause prominent disease symptoms when surface-inoculated [14]. On the other hand, *ost1-2* plants remained as resistant as wild-type L*er* plants to the nonpathogenic *hrcC* mutant (defective in the T3SS) of *Pst* DC3000 (Figure S2), indicating that the greatly enhanced susceptibility of *ost1-2* plants was specific to *Pst* DC3118.

For large-scale mutant screens we needed to grow plants in high density (approximately 20 to 30 plants per pot and 15 pots per flat). Accordingly, we tested whether *ost1-2* mutant plants would still exhibit enhanced susceptibility to *Pst* DC3118 under this growth condition. Indeed, when dip-inoculated with *Pst* DC3118, *ost1-2* plants allowed much higher bacterial multiplication and exhibited much more severe disease symptoms than wild-type L*er* plants (Figure S2). In subsequent mutant screening, *ost1-2* plants were included in each flat as a positive control.

### Identification and characterization of *scord* mutants

**Figure 1:**
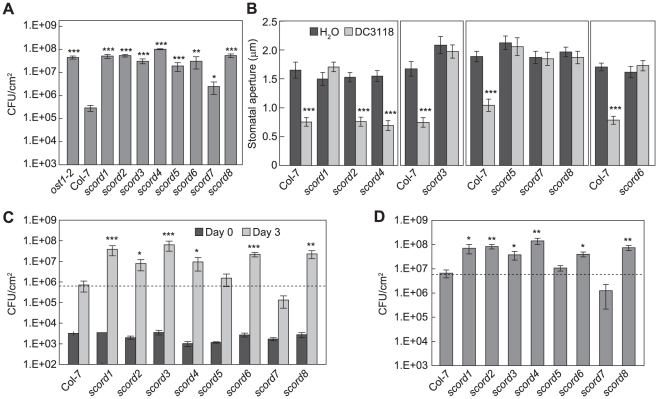
Characterization of *scord* mutants. (**A**) *Pst* DC3118 population at 3 dpi after dip-inoculation at 1×10^8^ CFU/ml. (**B**) Stomata apertures (µm) from leaf peels incubated with *Pst* DC3118 (1×10^8^ CFU/ml) for 1 h. (**C**) *Pst* DC3118 populations when inoculated by infiltration at 1×10^6^ CFU/ml. (**D**) *Pst* DC3000 population at 3 dpi after dip-inoculation at 1×10^7^ CFU/ml. Differences were detected by comparing each *scord* mutant with Col-7 for (**A**), (**C**) and (**D**) Statistical analyses for this and following figures are described in MATERIALS and METHODS.

About 14,000 activation-tagged T-DNA lines were screened by dip-inoculation with 1×10^8^ CFU/ml of *Pst* DC3118. Plants that showed disease symptoms similar to *ost1-2* plants were selected as putative mutants and allowed to set seeds. The progeny of each putative mutant were screened at least four more times in the following two generations. Here we report eight mutants that reproducibly showed enhanced disease symptoms, and are named *susceptible to coronatine-deficient Pst DC3000* (*scord*). *Pst* DC3118 populations in these mutants were almost 100 times higher than in wild-type plants when dip-inoculated, except for the *scord7* mutant, in which *Pst* DC3118 population was about 10 times higher (Figure 1A, Table 1).

**Table 1:** Phenotype summary of *scord* mutants.

	Col-7	*scord1*	*scord2*	*scord3*	*scord4*	*scord5*	*scord6*	*scord7*	*scord8*
**stomatal response to DC3118**	closure	open	closure	open	closure	open	open	open	open
**log(cfu/cm^2^), 3 dpi, DC3118 dipping (1E+8 cfu/ml)**	5.36±0.21	7.68±0.09	7.72±0.06	7.45±0.11	8.00±0.02	7.13±0.19	7.09±0.55	6.01±0.35	7.72±0.09
**log(cfu/cm^2^), 3 dpi, DC3118 infiltration (1E+6 cfu/ml)**	5.70±0.20	7.45±0.18	6.69±0.24	7.64±0.22	6.71±0.26	5.97±0.24	7.29±0.13	4.84±0.31	7.24±0.19
**log(cfu/cm^2^), 3 dpi, DC3000 dipping (1E+7 cfu/ml)**	6.68±0.23	7.74±0.18	7.89±0.09	7.47±0.18	8.10±0.12	6.98±0.12	7.57±0.09	5.65±0.33	7.84±0.09
**endogenous SA** a	++++	+	++++	−	++++	++++	++	++++	+
**DC3000-induced SA** a **(12 hpi, infiltration at 1E+8 cfu/ml)**	++++	−	++++	−	++++	++++	+++	++++	+++
**stomatal response to SA**	closure	closure	closure	closure	closure	closure	open	open	open
**stomatal response to ABA**	closure	closure	closure	closure	closure	closure	closure	open	closure
**rosette size**	++++	++++	+++++	+++++	++++	+++	++++	++++	+++
**leaf morphology**	normal	normal	normal	normal	serrated edge	pale green	smooth edge	thin	serrated edge
**stomatal density**	normal	normal	normal	normal	normal	normal	normal	normal	normal
**stomatal morphology**	normal	normal	normal	normal	normal	normal	large and missing central ridges	normal	normal
**gene/chromosome**				At4g39030/IV		At1g64550/I	18.84 Mb–19.03 Mb/III	1.9 Mb–2.14 Mb/V	

a SA levels from *scord* mutants were compared to those from Col-7, based on Figure 2.

As the *scord* mutants may be affected in different aspects of COR action during *Pst* DC3000 infection, we first determined whether some of them are defective in the bacterium-induced stomatal closure response. When subjected to leaf peel assays with suspensions of *Pst* DC3118 [14], [19], most stomata of wild-type Col-7 plants closed and resulted in a significant reduction of the mean aperture (Figure 1B). However, the stomata of six *scord* mutants—*scord1*, *-3*, *-5*, *-6*, *-7*, and -*8*—did not show significant closure, indicating loss of normal stomatal closure response to *Pst* DC3118 (Figure 1B). The other two mutants, *scord2* and *scord4*, maintained normal stomatal closure responses to bacteria, at least within the time frame of our assays (i.e., 1 to 1.5 h after incubation) (Figure 1B).

Since increased plant susceptibility in dip-inoculation experiments could also be caused by a deficiency in apoplastic defense after bacterial invasion, we next tested *scord* mutants by infiltration inoculation with *Pst* DC3118 (Figure 1C). Of the eight *scord* mutants, *scord1*, -3, -*6*, and -*8* plants showed a much higher apoplastic susceptibility to *Pst* DC3118, and contained 50 to 100 times more bacteria 3 days after inoculation, compared to Col-7 plants. *scord2* and *scord4* also showed a higher susceptibility to *Pst* DC3118 in infiltration experiments, harboring about 10 times more bacteria than Col-7 plants at day 3 (Figure 1C). In contrast, *scord5* and *scord7* did not show significantly higher susceptibility to *Pst* DC3118 in infiltration experiments compared to Col-7 plants (Figure 1C). Although statistically (Student's t-test) not significant, we have consistently observed in all repeats that *Pst* DC3118 growth is actually less in *scord7* plants than in Col-7 plants. Overall, these results suggest that *scord1*, -*3*, *-6*, and *-8* are deficient not only in stomatal defense but also in apoplastic defense. On the other hand, *scord5* and *scord7* are deficient in stomatal defense, and *scord2* and *scord4* are affected in apoplastic defense.

Next, we tested the susceptibility of the *scord* mutants to wild type *Pst* DC3000 by dipping inoculation. We found that *scord1, -2, -3, -4, -6, and -8* mutants were also more susceptible to *Pst* DC3000 than Col-7 plants (Figure 1D). In contrast, *scord5* and *scord7* were not hyper-susceptible to *Pst* DC3000 compared to Col-7 plants. Again, although statistically not significant, *Pst* DC3000 growth was consistently less in *scord7* plants than in Col-7 plants.

Finally, we examined the susceptibility of these eight *scord* mutants to the nonpathogenic *Pst* DC3000 *hrcC* mutant by dipping inoculation. None of the eight *scord* mutants allowed significant growth of *Pst* DC3000 *hrcC* (Figure S3). Thus, *scord* mutants maintained normal resistance to nonpathogenic bacteria.

### Effects of *scord* mutations on SA accumulation and/or SA-induced stomatal closure

We have previously shown that SA plays a critical role in bacterium-induced stomatal closure response [14], [19]. To position the *scord* mutants in relation to SA, we tested SA-induced stomatal closure in the *scord* mutants (Figure 2). In leaf peel assays, we found that *scord6*, -*7* and -*8* mutants are defective in SA-induced stomatal closure, but *scord1*, -*2*, -*3*, -*4* and -*5* had normal stomatal closure response, similar to wild type Col-7 plants.

**Figure 2:**
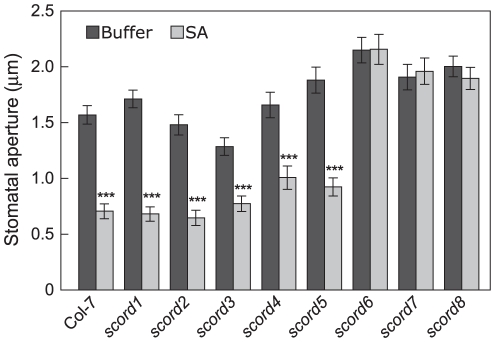
Stomatal closure responses of *scord* mutants to SA. Stomatal apertures (µm) were measured from leaf peels incubated with SA (100 µM) for 1 h.

**Figure 3:**
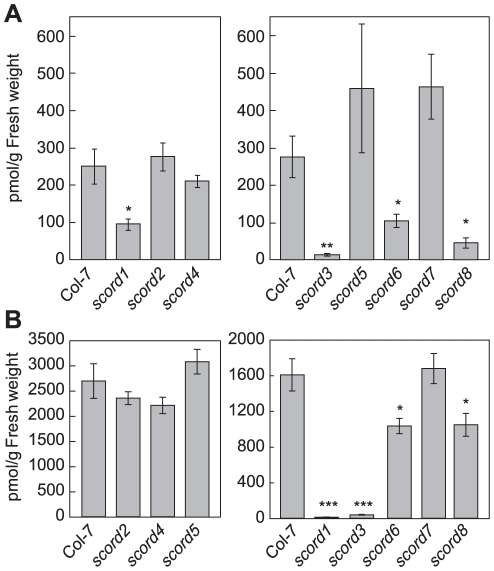
Salicylic acid (SA) levels in leaves of Col-7 and *scord* mutants. Numbers represent means and standard errors of values from 4 different plants. Similar results were obtained from two different experiments. (**A**) Plants without treatment. (**B**) Plants were infiltrated with *Pst* DC3000 at 1×10^8^ CFU/ml. Leaves were collected at 12 hpi.

We conducted further experiments to examine whether any of the *scord* mutants were affected in SA accumulation. We found that *scord1*, -*3*, -*6*, and -*8* plants all had significantly lower basal levels of SA, containing only 10–30% of what was detected in wild-type Col-7 plants (Figure 3A). To the contrary, SA levels in *scord2*, *-4*, -5, and -*7* plants were similar to those in wild-type Col-7 plants (Figure 3A).

We also examined *scord* mutants for SA levels 12 hours after infiltration with *Pst* DC3000 at 1×10^8^ CFU/cm (Figure 3B). Similar to what was observed without bacterium challenge, the SA levels in *scord1*, -*3*, -*6*, and -*8* plants were reduced, whereas those of *scord2*, -*4*, -*5*, and -*7* plant were similar to those in Col-7 plants (Figure 3B).

### Effects of *scord* mutations on ABA-induced stomatal closure

**Figure 4:**
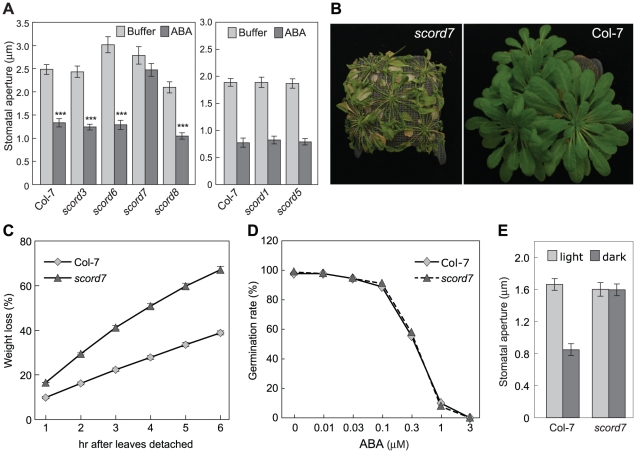
Responses of *scord* mutants to ABA. (**A**) Stomatal apertures of Col-7 and *scord1*, -*3*, -*5*, -*6*, -7 and -8 in response to ABA (10 µM) in leaf peel assay. (**B**) Appearance of 5-week-old *scord7* and Col-7 plants after being withheld water for 10 days. (**C**) Water loss of detached leaves. Four plants and 4 to 5 leaves from each plant were sampled for Col-7 or *scord7*. Values are means with standard errors displayed. (**D**) Germination rates of Col-7 and *scord7* seeds on MS plates supplemented with ABA. Values are means with standard errors. (**E**) Stomata apertures of Col-7 and *scord7* in response to dark. Leaf peels were incubated in buffer under light or in the dark for 3 h before stomatal aperture measurements.

Besides SA, we have previously shown that ABA biosynthesis and signaling are also critical for the stomatal closure response to bacteria or MAMPs [14], [19]. To position the six stomata-closure-defective *scord* mutations in relation to ABA, we examined the stomatal response of *scord1*, *-3*, *-5*, *-6*, -*7*, and *-8* mutants to ABA. We found that only *scord7* stomata failed to close in response to ABA (Figure 4A), whereas stomata of Col-7 and the other five *scord* mutants (*scord1*, *-3*, *-5*, *-6*, and *-8*) showed normal closure responses to ABA (Figure 4A), suggesting that the *scord7* mutant might be affected in ABA response or a general step in stomatal closure that is common to multiple stomatal closure pathways. ABA response mutants often show hypersensitivity to drought stress. Indeed, when subjected to water withholding, *scord7* plants wilted faster than wild-type Col-7 plants (Figure 4B). When detached leaves were tested for water loss, leaves from *scord7* plants lost water at a much faster rate than those from Col-7 wild type plants (Figure 4C). The *scord7* mutant, however, showed a similar ABA inhibition response as wild type Col-7 in seed germination assay (Figure 4D). We also tested *scord7* plants for dark-induced stomatal response, which is thought to be independent of ABA signaling [30]. The *scord7* plants were defective in dark-induced stomatal closure (Figure 4E). Thus, SCORD7 is involved either in a guard cell-specific ABA response pathway or in a later step shared by SA-, ABA- and dark signaling pathways leading to stomatal closure.

### Morphological phenotypes of *scord* mutants

**Figure 5:**
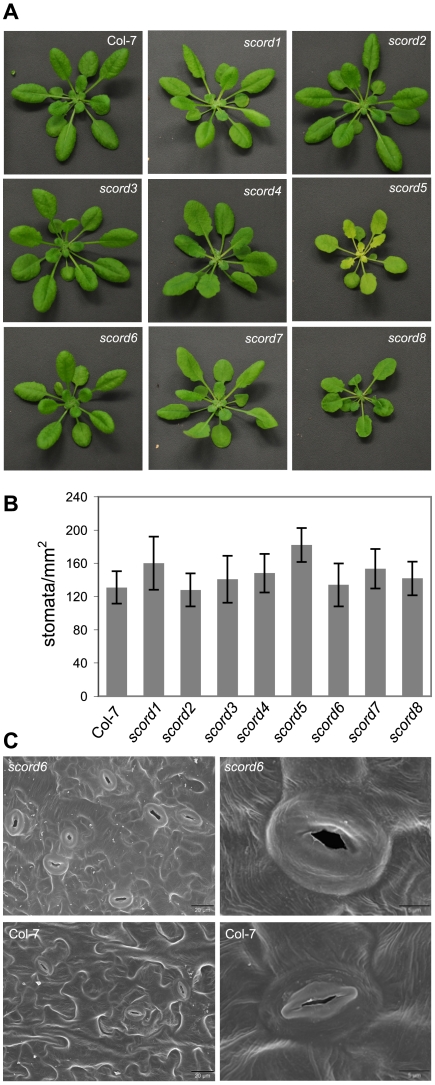
Morphological phenotypes of *scord* mutants of 4 to 5 weeks old. (**A**) Rosettes. (**B**) Stomata densities of leaf abaxial surfaces. (**C**) Scanning electron microscopy pictures of leaf abaxial surfaces of Col-7 and *scord6* plants. Note that raised central ridges in stomata are missing in the *scord6* mutant.

In addition to the phenotypes characterized above, we also noticed morphological phenotypes for some of the *scord* mutants. Among the eight mutants, the appearances of *scord1*, -*2*, -*3*, and -*7* plants are similar to wild type Col-7 plants (Figure 5; Table 1). On the other hand, compared to Col-7 plants, *scord5* and *8* plants have slightly smaller rosettes, *scord5* plants have pale green leaves, *scord4* and *8* plants have more serrated leaf edges, and *scord6* has smoother leaf edges (Figure 5A). We also examined the stomata density and stomata morphology in these mutants, and did not notice any dramatic difference on stomata density (Figure 5B), except that *scord6* stomata appear to be larger and lack the central ridges (Figure 5C). At present, it is not known whether these morphological phenotypes are caused by the same mutations that lead to defects in stomatal closure and/or bacterial susceptibility phenotypes.

### The *scord3* and *scord5* mutants carry a T-DNA insertion in the *EDS5* gene and *AtGCN20/AtABCF3* gene, respectively

We attempted plasmid rescue experiments with all *scord* mutants. However, we were able to correlate a T-DNA insertion with mutant phenotypes in only the *scord3* and *scord5* mutants. In the *scord3* mutant, the T-DNA insertion is located in the first intron of At4g39030 (Figure S4), which encodes EDS5, a putative transporter protein required for SA biosynthesis [31]. Similar to the *scord3* mutant, the *eds5-1* mutant showed a defect in SA accumulation and stomatal response (Figure S5). Furthermore, allelism tests showed that *scord3* and *eds5-1* have mutations in the same gene (Figure S6). In the literature, three alleles of *eds5* have been characterized [31]. We therefore have named *scord3 eds5-4*.

As described above, in addition to the *scord3/eds5-4* mutant, the *scord1* mutant also contained little SA before or after bacteria challenge (Figure 2). Like the *scord3/eds5-4* mutant, the *scord1*mutant maintained a normal SA-induced stomatal closure response, suggesting that the *scord1* mutant is deficient in the synthesis of SA, but is normal in SA signaling. We therefore examined whether the *scord1* mutant also contains a mutation in the *EDS5* gene. We amplified and sequenced the genomic region of *EDS5* from *scord1* plants. However, we did not find any mutation in *EDS5* (data not shown). Therefore, the *scord1* mutant likely has a mutation in a gene, other than *EDS5*, that is involved in SA biosynthesis.

**Figure 6:**
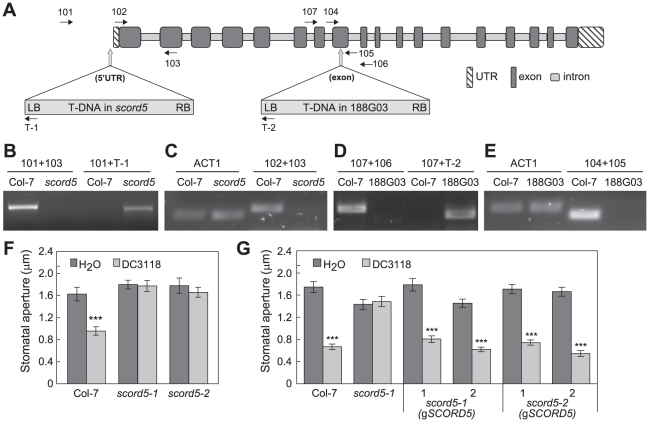
Characterization of *SCORD5* (At1g64550). (**A**) The intron/exon structure of At1g64550 and T-DNA insertion sites in *scord5* (*scord5-1*) and 188G03 (*scord5-2*) mutants. Locations of primers used in this study are indicated. (**B**) and (**D**) PCR reactions using genomic DNA from Col-7 and *scord5* (*scord5-1*) (**B**) or 188G03 (*scord5-2*) (**D**) as template. (**C**) and (**E**) RT-PCR reactions using total RNA from Col-7 and *scord5* (*scord5-1*) (**C**) or 188G03 (*scord5-2*) (**E**) as template. The RT-PCR product of *ACT1* (Arabidopsis actin 1; At2g37620) was used as a loading control. (**F**) Stomata apertures of Col-0, *scord5-1*, and *scord5-2* leaf peels incubated with H_2_O or *Pst* DC3118 (1×10^8^ CFU/ml) for 1 h. (**G**) Stomata apertures of Col-7, *scord5-1* and *scord5* mutant plants complemented with a 5.4-kb-genomic region of At1g64550, in response to *Pst* DC3118 (1×10^8^ CFU/ml) for 1 h.

The T-DNA insertion site in the *scord5* mutant lies in the 5′UTR region of At1g64550 (108 bps upstream of the start codon ATG; Figure 6A). The existence of this T-DNA insertion in the *scord5* genome was confirmed by PCR using At1g64550-specific and T-DNA-specific primers (Figure 6B). Furthermore, the transcript of At1g64550 was not detectable in *scord5* plants (Figure 6C). To confirm that the phenotypes observed in *scord5* plants were due to the T-DNA insertion in At1g64550, we identified an independent T-DNA insertion line, 188G03, in the GABI-Kat collection [32]. Genomic PCR confirmed the presence of a T-DNA insertion in the eighth exon of At1g64550 (Figure 6A, D). RT-PCR using primers near the insertion site showed that 188G03 is also a knockout line for At1g64550 (Figure 6E). Like the *scord5* mutant, 188G03 was also impaired in stomatal closure response to *Pst* DC3118 (Figure 6F). Based on these molecular and phenotypic analyses, we have designated the GABI-Kat line 188G03 as *scord5-2* and the original *scord5* line as *scord5-1*.

To complement the phenotypes of *scord5* mutants, a 5.4-kb genomic fragment that includes a 480-bp region upstream of the start codon and a 400-bp region downstream of the stop codon of *SCORD5* (At1g64550) was amplified using Col-7 genomic DNA. This *SCORD5* fragment was cloned into the plant transformation vector pCAMBIA1300 and introduced into both *scord5-1* and *scord5-2* mutant plants via *Agrobacterium*-mediated transformation. Examination of four randomly chosen, independent T1 transformants showed that stomatal closure response to *Pst* DC3118 was restored in both *scord5-1* (g*SCORD5*) and *scord5-2* (g*SCORD5*) lines (Figure 6G), providing further evidence that *SCORD5* is required for bacterium-triggered stomatal closure.

Sequence analysis showed that *SCORD5* encodes a gene that has been annotated as AtGCN20/AtABCF3, which belongs to the F subfamily of ATP-binding cassette (ABC) proteins. *AtGCN20/AtABCF3* is a 4.8-kb gene with 17 introns, and encodes a protein of 715 amino acids. AtGCN20/AtABCF3 (*SCORD5* hereinafter) is predicted to have only nucleotide-binding domains (NBDs), lacking transmembrane domains that are characteristic of the AtABCA, -B, -C, -D, and -G protein subfamilies [33]–[35].

### Molecular characterizations of other *scord* mutations

Because T-DNA insertions in other *scord* mutants (i.e., other than *scord 3* and *scord5*) either could not be amplified, or were amplified but not linked to *Pst* DC3118-hypersusceptibility phenotypes (data not shown), we have initiated physical mapping of two of these *scord* mutations: *scord6* and *scord7*. To this end, we have narrowed the *scord6* mutation to a 190-kb region on chromosome III, and the *scord7* mutation to a 240-kb region on chromosome V (Table 1). Neither region contains genes that are known to be involved in MAMP, SA or ABA signaling or in stomatal closure response. Therefore, *SCORD6* and *SCORD7* likely encode new regulators of stomatal closure response.

In summary, our molecular and phenotype characterization of the eight *scord* mutants suggests that *scord3*, -*5*, -*6*, and -*7* represent mutations in four different genes (i.e., *EDS5*, *AtGCN20/AtABCF3*, *SCORD6*, and *SCORD7*). Although we cannot ascertain the genetic relationships among the other four *scord* mutations (*scord1*, *-2*, *-4*, and *-8*), the distinct phenotypes (stomatal closure response, apoplast defense, SA levels, and morphology; Table 1) exhibited in the corresponding mutants suggest that at least some of them would be affected in additional genes.

### The *scord5* mutant is affected in MAMP-induced stomatal closure, but not other MAMP-induced responses in the leaves

**Figure 7:**
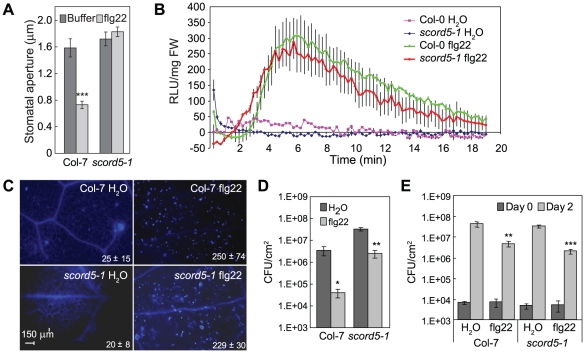
*scord5* mutant plants have normal flg22-induced responses other than stomatal closure response. (**A**) Stomata apertures of Col-7 and *scord5-1* leaf peels treated with flg22 (10 µM). (**B**) H_2_O_2_-dependent luminescence of luminol induced by flg22 (100 nM) in Col-7 and *scord5-1*seedlings. (**C**) Callose deposition induced by flg22 (10 nM) in Col-7 and *scord5-1* leaves. (**D**) and (**E**) Populations of *Pst* DC3000 at 2 dpi after inoculation with dipping (**D**; 1×10^8^ CFU/ml) or infiltration (**E**; 1×10^6^ CFU/ml) following spray with H_2_O or flg22 (3 µM) a day earlier. Differences were detected by comparing H_2_O and flg22 treatment. Experiments in (**B**) and (**C**) were repeated two and three times, respectively.

Because *SCORD5* has never been implicated in stomatal closure or plant defense response, we focused on this gene for further analysis. We recently showed that the signaling cascade for bacterium/MAMP-induced stomata closure involves MAMP signaling, followed by SA and ABA signaling [19], [36]. As stomata of the *scord5* mutant responded normally to SA and ABA (Figures 2, 4), we investigated a possible defect in MAMP response using flg22 (a bioactive 22-aa peptide from bacterial flagellin). We found flg22 triggered stomatal closure in Col-7 plants, but not in *scord5* plants (Figure 7A). Similarly, elf18 (a bioactive 18-aa peptide from *E. coli* EF-Tu) induced stomatal closure in Col-7 plants, but not in *scord5* plants (Figure S7). These results are consistent with the observed defect in stomatal closure response to *Pst* DC3118 bacteria (Figure 1B), which are expected to produce flagellin and possibly other MAMPs during infection [19].

Stomatal closure is only one of several functional outputs induced by MAMPs. To determine whether other responses induced by MAMPs are also affected in the *scord5* mutant, we first examined the production of reactive oxygen species and deposition of callose in *scord5* leaves in response to flg22. The production of H_2_O_2_ after flg22 treatment (100 nM) was similar in *scord5-1* and Col-7 plants (Figure 7B). Similarly, the amounts of callose deposition induced by infiltration of flg22 (10 nM) were comparable in *scord5-1* and Col-7 leaves (Figure 7C). Next, we studied the effect of flg22 pretreatment on the induced resistance against *Pst* DC3000 (i.e., flg22 protection assay) in *scord5-1* and Col-7 plants. One day after spraying *scord5-1* and Col-7 plants with 3 µM flg22, we inoculated plants with *Pst* DC3000 by dipping at 1×10^8^CFU/ml (Figure 7D). We noticed that *scord5* plants were more susceptible than Col-7 to *Pst* DC3000 in the H_2_O treatment control, and allowed more *Pst* DC3000 multiplication, compared to Col-7 after flg22 pretreatment (Figure 7D). Nonetheless, the flg22 treatment still induced resistance in *scord5* plants, albeit to a smaller degree compared to that in Col-7 plants. To find out whether the higher susceptibility of the *scord5* mutant shown in the flg22 protection assay in dipping inoculation was caused by reduced apoplastic defense, we repeated the flg22 protection experiment by infiltrating *Pst* DC3000 (1×10^6^ CFU/ml) directly into the apoplast. We found that the multiplication of *Pst* DC3000 was suppressed to similar levels in *scord5-1* and Col-7 leaves (Figure 7E). These experiments suggest that flg22-induced apoplastic defense is normal in the *scord5* mutant and that the partial loss of flg22-induced resistance in *scord5* plants is likely caused by a defect in bacterium-triggered stomatal closure.

### ILITYHIA (ILA) is also required for bacterium-triggered stomatal closure response

**Figure 8:**
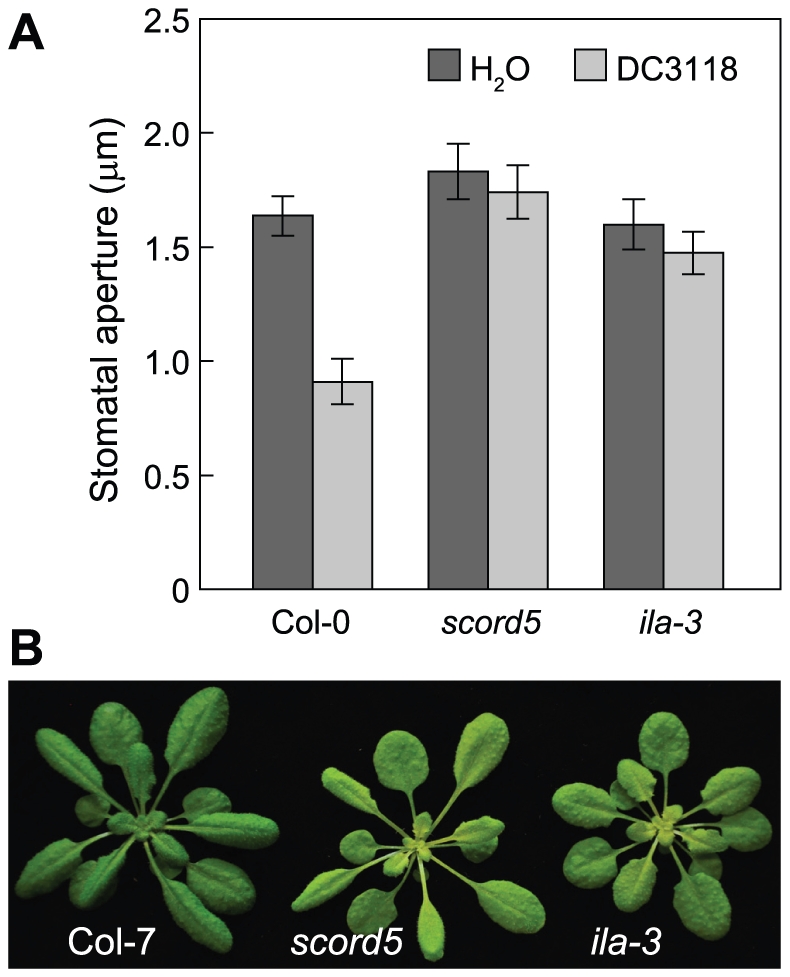
Stomatal closure response in the *ila-3* mutant. (**A**) Stomata apertures of Col-7, *scord5* and *ila-3* in response to *Pst* DC3118 (1×10^8^ CFU/ml) at 1 hpi. (**B**) Four-week-old rosettes.

We conducted further experiments to increase our understanding of the involvement of the SCORD5-associated pathway in regulating bacterium-triggered stomatal closure. SCORD5 shares significant sequence similarities with yeast (46% identity and 66% similarity) and mammalian (41% identity and 61% similarity) GCN20 proteins (EDN59158 and NP_038880, respectively), which, together with GCN1, regulates stress-associated protein translation [37]–[39]. Arabidopsis has a putative GCN1 orthologue named ILITYHIA (ILA; At1g64790), which was shown recently to be involved in defense against *Pst* DC3000 [40]. Arabidopsis ILA shares 35% identity and 54% similarity with rat GCN1 (NP_001162135) and 34% identity and 54% similarity with yeast GCN1 (EEU04663), but shares no sequence similarity with SCORD5 (AtGCN20/AtABCF3). We examined the possibility that ILA may also be required for bacterium-triggered stomatal closure. Indeed, similar to the *scord5* mutant, stomata of *ila-3* mutant were completely unresponsive to *Pst* DC3118 (Figure 8A). This result provides further evidence that bacterium-triggered stomatal closure is likely regulated by an authentic GCN1/GCN20-associated protein translation control mechanism in plants.

## Discussion

In the Arabidopsis-*Pst* DC3000 interaction, COR has been implicated in several aspects of pathogenesis. To date, however, a random genetic screen for host mutants that rescue the virulence of COR-deficient *Pst* mutants has not been reported. In principle, such a genetic screen not only could identify new alleles of host genes known to be involved in COR-dependent interactions during infection, but also has the potential to uncover novel components in the host that are required for the various virulence functions of COR. Indeed, from an initial screen of ∼14,000 T-DNA insertion lines, we were able to isolate eight *scord* mutants. Further detailed characterization of these mutants uncovered both known (e.g., EDS5) and novel (e.g., AtGCN20/AtABCF3, and possibly SCORD6 and SCORD7) regulators of stomatal and/or apoplastic defenses, illustrating the exciting prospect of future large-scale *Pst* DC3118-based genetic screens in the identification of cell-type-specific defense mutants in plants.

Stomatal closure response has been shown to be an integral part of Arabidopsis resistance to COR-deficient mutants of *Pst* DC3000, and SA is necessary for this response [14], [19]. Indeed, of the eight *scord* mutants isolated in this study, six are defective in the stomatal closure response to *Pst* DC3118 (Figure 1B). Among them, both basal and bacterium-induced levels of SA are significantly lower in *scord1*, -*3*, -*6* and -*8* plants than those in wild-type plants (Figure 3), indicating that these four mutants have defects in the accumulation of SA to various degrees. The *scord7* mutant is defective in stomatal closure response to SA, ABA and dark, and is hypersensitive to drought (Figure 4). These results point to a possible deficiency in a step common to multiple signaling pathways leading to stomatal closure in the *scord7* mutant plant.

SA is also known to be essential for apoplast defense, which can be revealed using infiltration inoculation. In our screen, SA-compromised *scord1*,*-3*, -*6* and -*8* mutants allowed more bacterial growth, compared with wild-type plants, when *Pst* DC3118 was infiltrated into the apoplast directly (Figure 1C), supporting the importance of SA in mediating the apoplastic defense against *Pst* DC3118. In contrast, although the *scord7* mutant is more susceptible than Col-7 plants to surface-inoculated DC3118, this mutant showed slightly more resistance to *Pst* DC3118 in infiltration experiments (Figure 1C). This is a particularly interesting result because ABA mutants have been observed to be more resistant to *Pst* DC3000 in infiltration experiments [41]. The enhanced apoplastic resistance of the *scord7* mutant to *Pst* DC3118 therefore could be related to the inability of *scord7* stomata to respond to ABA. However, considering that *scord7* stomata are not responsive to SA and dark, we cannot rule out the possibility that the apoplast of the *scord7* mutant has elevated resistance due to an ABA signaling-independent mechanism. Regardless, the overall higher level of susceptibility of the *scord7* mutant to surface-inoculated *Pst* DC3118 can now be explained by a combined effect of compromised stomatal defense and enhanced apoplastic defense, with the stomatal closure defect overpowering the enhanced apoplastic defense in this mutant. We noted that the overall enhancement of *Pst* DC3118 growth in the *scord7* mutant when inoculated by dipping was less than in other *scord* mutants (Figure 1A), which is consistent with the observation that the apoplast of other *scord* mutants was not more resistant to *Pst* DC3118, as revealed in the infiltration experiments (Figure 1C).

It is interesting that, although the *scord5* and *scord7* mutants are much more susceptible to surface-inoculated *Pst* DC3118, compared with Col-7 plants, they are not more susceptible to surface-inoculated *Pst* DC3000. Thus, the disease hyper-susceptibility of the *scord5* and *scord7* mutants is dependent on COR production in bacteria. This finding is consistent with the observations that stomatal defense in wild-type Col-7 plants presents a significant barrier to infection by *Pst* DC3118 and that the *scord5* and *scord7* mutants are defective in stomatal defense, thereby allowing *Pst* DC3118 to infect from the leaf surface. On the other hand, *Pst* DC3000 can infect not only *scord5* and *scord7* mutants, but also wild-type Col-7 plants because of its ability to produce COR, which counteracts stomatal closure in wild-type Col-7 plants. COR is not necessary for *Pst* DC3000 to infect *scord5* and *scord7* mutants, in which stomatal closure is already compromised.

In this study, we also identified two mutants, *scord2* and *scord4*, that showed an apparently normal stomatal closure response, but compromised apoplastic defense (Figure 1). Interestingly, in contrast to *scord5* and *scord7* mutants, *scord2* and *scord4* mutants were hyper-susceptible to both *Pst* DC3118 and *Pst* DC3000, suggesting that these two mutants have a general defect in the apoplast (defense or nutrition) and that the hyper-susceptibility phenotype is not dependent on COR production in bacteria. The ability of these mutants to rescue the virulence of *Pst* DC3118 suggests that a hyper-susceptible apoplast can compensate, at least genetically, for the reduced ability of *Pst* DC3118 to overcome stomatal defense. We hypothesize that these apoplast defense mutants are likely affected in cellular processes other than the biosynthesis or signaling of SA and ABA, because defects in SA or ABA biosynthesis or signaling cause altered stomatal closure in response to bacteria [14], [19] and would have been detected in this study. Accordingly, further characterization of the *scord2* and *scord4* mutants has potential to yield novel genes/pathways that control apoplast defense (or nutrition) in Arabidopsis.

We were successful in cloning *SCORD3* and *SCORD5*. Whereas *SCORD3* turns out to be *EDS5*, which is known to be involved in plant defense, *SCORD5* is a new component required for bacterium-triggered stomata closure response. The *scord5* mutation affected mainly MAMP-induced stomatal closure, but not SA- or ABA-induced stomatal closure, suggesting that SCORD5 likely acts early in the stomatal closure response pathway. Although we could not detect a significant defect in apoplastic defense in the *scord5* mutant, the *ila-3* mutant has been shown to be more susceptible to *Pst* DC3000 compared with Col-0 plants by infiltration inoculation, suggesting a defect in apoplastic defense [40]. We noticed that the *ila-3* plants are slightly smaller than *scord5* plants (Figure 8B). Because *SCORD5* belongs to a five-member gene family (Figure S8), whereas there is only a single *ILA* gene in Arabidopsis, we speculate that some of the *SCORD5*-family genes may have partially redundant functions and that one or more of *SCORD5*-like genes may compensate for the loss of *SCORD5* in the mesophyll cells. Future research should determine whether higher-order mutants of the *SCORD5* family genes would phenocopy the pleiotropic defects in both stomatal and apoplastic defenses in the *ila-3* mutant.

Overall, the genetic screen reported in this paper illustrates an unbiased and useful approach in the understanding of Arabidopsis signaling pathways that govern cell-type-specific (i.e., stomatal and mesophyll) responses to *Pst* DC3000 infection, as well as the molecular action of COR during various stages of *Pst* DC3000 infection of Arabidopsis.

## Materials and Methods

Unless specified, all experiments presented were repeated three times and statistical differences were detected with a two-tailed T-test (*, P<0.05; **, P<0.01; ***, P<0.001).

### Plant materials and growth conditions

Arabidopsis plants were grown in soil under a 12/12 h photoperiod at 110 µmol m^−2^ s^−1^ at 22°C. Seeds of the activation-tagged T-DNA lines (CS21995, CS23153) were purchased from the Arabidopsis Biological Resource Center with Col-7 accession as the wild-type parent [42]. For ABA inhibition of germination, seeds are sterilized in 30% bleach for 15 min and washed in sterilized ddH2O for 5 times before put on MS (1% sucrose) plates. Ninety seeds were used for each treatment. Plates were left at 4°C for two days before placed in growth chambers, and the numbers of seeds germinated were recorded two days later. For stomata counting, three leaves were chosen from 5-week old plants. A middle section of each leaf was cut out and mounted in distilled water, and visualized with bright field microscopy. From each section, three areas of 0.45 µm^2^ were chosen for observation. The chosen areas were scanned to a depth of 40–60 µm with a Zeiss 510 Meta ConfoCor3 laser scanning confocal microscope (Carl Zeiss MicroImaging, Thornwood, NY) to generate Z-scans. Zeiss LSM software was used to assemble the z-series into stacked images representing the leaf surface for stomata counting.

### Stomata assays

Stomatal assays followed procedures described in a previous study [19]. Leaf peels from the abaxial side were collected from mature leaves of 5-week-old plants and placed in 250–300 µl of ddH_2_O or bacteria resuspended in ddH_2_O (1×10^8^ CFU/ml), buffer (25 mM MES, 10 mM KCl, pH 6.15) or buffer containing SA or ABA (Sigma, St. Louis, MO, USA), flg22 or elf18 (EZBiolab, Westfield, IN, USA) on glass slides in square Petri dishes with lids on. To preserve the stomatal aperture status in plants used for both stomatal and bacterial pathogenesis assays, we did not further treat leaf peels in any stomatal opening buffer. The Petri dishes were left for an hour in the growth chamber in which plants were grown before being viewed under the microscope. Leaf peels on slides were observed under a light microscope and images were randomly taken to ensure that at least 30 stomata were recorded for each sample treatment. Images were opened in Adobe Photoshop for measurement of stomatal apertures. Bacteria were used at 1×10^8^ CFU/ml (OD_600_ = 0.2). Stomatal apertures shown in results are means and standard errors of 30–60 measurements for each experiment.

### Bacterial infection assays

A 10-ml low-salt LB liquid culture was started using bacteria (*Pst* DC3118) scraped from a plate that had been stored at −4°C for less than two weeks. After 12 h at 28°C, a larger subculture was started using 1∶100 dilution at 28°C. Bacterial cells were harvested when the OD_600_ of the culture reached 0.8 to 1.0. Bacteria were resuspended in ddH_2_O to an OD_600_ of 0.2 (1×10^8^ CFU/ml). Silwet L-77 was added to a final concentration of 0.02 to 0.05%. For DC3118 dipping screening, four-week-old plants grown in meshed pots, at a density of about 20 seedlings per pot, were dipped upside down in the bacterial solution for a few seconds to coat all leaves uniformly with bacterial suspension. Plants were returned to a growth room in a tray covered with a plastic lid. At day 3 post-inoculation (3 dpi), plants showing no disease symptoms were removed from the pot. Diseased plants were allowed to set seeds. Seeds from the putative mutant plants were sown and the screening process was repeated. For other bacterial infection assays via dipping or infiltration inoculation, disease symptoms were recorded by camera and bacterial populations were monitored by serial-dilution assays according to Katagiri et al. [3]. All bacteria growth results are recorded as means of four different leaves from four different plants, with standard errors indicated.

### flg22 protection assay

Five-week-old Arabidopsis plants were sprayed with H_2_O or 3 µM flg22 (EZBiolab, Carmel, IN) 24 h before bacterial inoculation. The flg22-treated plants were inoculated with *Pst* DC3000 by dipping at 1×10^8^ CFU/ml or infiltration at 1×10^6^ CFU/ml. Two days after bacterium inoculation, leaf samples were collected for bacterial enumeration.

### Plasmid rescue cloning of T-DNA flanking fragments from the *scord3* mutant

The plasmid rescue procedure was adapted from Weigel et al. [42]. Genomic DNA was prepared from 1 g (fresh weight) leaf tissue of 5-week-old plants using the Nucleon Phytopure Plant DNA Extraction Kit (Amersham Biosciences, Piscataway, NJ). One to five µg of purified genomic DNA was digested in a 120-µl reaction mixture overnight with restriction enzymes *Bam*HI, *Spe*I, and *Not*I for the left border and *Kpn*I, *Eco*RI, and *Hin*dIII for the right border of the T-DNA [42]. After digestion, ddH_2_O was added in the reaction mixture to bring the volume to 500 µl, followed with phenol-chloroform extraction and then chloroform extraction. Digested DNA was precipitated with 2 volumes of ethanol and 1/10 volume of sodium acetate (3 M, pH 5.2), and washed twice with 70% ethanol. Dried DNA was resuspended in 100 µl ddH_2_O, then used in a ligation reaction of 120 µl with 20–40 U of T4 DNA ligase (NEB, Beverly, MA) overnight at 16°C. DNA from the ligation mixture was precipitated in the same way as before and resuspended in 10 µl ddH_2_O. One to two µl of the ligation mixture was used for transformation into the SURE Electroporation-Competent Cells (Stratagene, La Jolla, CA). Plasmids from the Ap^r^ colonies were digested with the same restriction enzymes used for plasmid rescue reactions, and the inserts were subjected to sequencing using primers T3 (5′-ATTAACCCTCACTAAAGGGA-3′), T7 (5′-TAATACGACTCACTATAGGG-3′), T-1 (5′-AAGTGCAGGTCAAACCTTGAC-3′), T-3 (5′-GGTAATTACTCTTTCTTTTCCTC-3′), or sfEcoRI (5′-AGCCTTGCTTCCTATTATATCT-3′). Primers used to confirm the T-DNA insertion in *scord3/eds5-4* plants also include 108 (5′-AAGGAAGCTCCATCGAACT-3′) and 109 (5′-TGTTTGGCAAGAGAAGTAGCA-3′).

### iPCR cloning of T-DNA flanking fragments from the *scord5* mutant

Genomic DNA was prepared the same way as described for plasmid rescue. One µg of purified genomic DNA was digested in a 100-µl reaction mixture overnight with the restriction enzyme *Eco*RV, *Hin*dIII, *Dra*I, or *Eco*RI. After digestion, ddH_2_O was added to the reaction mixture to bring the volume to 500 µl, and the DNA mixtures were purified using a QIAquick PCR purification kit (Qiagen, Valencia, CA) and eluted in 50 µl ddH_2_O. Ten µl of the purified DNA mixture was incubated overnight at 16°C in a ligation reaction of 100 µl with 10 to 30 U of T4 DNA ligase (NEB, Beverly, MA). Five µl of the ligation mixture was used in each PCR reaction, along with the following primers specific to the T-DNA [42]: iPCR1 (5′-TGGATCTCAACAGCGGTAAGA-3′), iPCR2 (5′-TTCGACGTGTCTACATTCACG-3′), iPCR3 (5′-TCGGTGTGTCGTAGATACTAG-3′), iPCR4 (5′-TGGTTGACGATGGTGCAGAC-3′), iPCR1g (5′-TGACCATCATACTCATTGCTGATCC-3′), iPCR2g (5′-CGATCCGTCGTATTTATAGGCGAAAG-3′), T-1 (5′-AAGTGCAGGTCAAACCTTGAC-3′), T-3 (5′-GGTAATTACTCTTTCTTTTCCTC-3′), and T7 (5′-TAATACGACTCACTATAGGG-3′). PCR products obtained were purified from agarose gels after electrophoresis and sequenced. The T-DNA flanking sequence was identified from a PCR fragment amplified using primers T-3 and iPCR3 and *Dra*I-digested *scord5* genomic DNA as template.

### Gene cloning

The primers used for PCR to confirm the T-DNA insertions in *scord5-1* and *scord5-2* plants, and for RT-PCR to confirm lack of expression of *SCORD5* in the mutant plants were as follows: 101 (5′-CAAGTTATAGATAATATGAGTTTGT-3′), 102 (5′-GAATCTCGTCGCTTCTGTGTT-3′), 103 (5′-AACTTATCCGACAGTTTGCTAC-3′), 104 (5′-GTGGTCACGGACATCATCCAT-3′), 105 (5′-CTGCATATGTGACCGTGATCG-3′), 106 (5′-CAAACCAGAGACGAGTTGGAAACAG-3′), 107 (5′-CAGGTTTCTGACATTCTTTTCCTC-3′), T-1 (5′-AAGTGCAGGTCAAACCTTGAC-3′), T-2 (5′-ATATTGACCATCATACTCATTGC-3′), ACT1 (5′-GGTCGTACTACCGGTATTGTGCT-3′; 5′-TGACAATTTCACGCTCTGCTGTG-3′).

The 5.4-kb genomic fragment of *SCORD5*/*At1g64550* was amplified using the genomic DNA of Col-7 as template. This fragment contains 480 bps upstream of the start codon ATG with an *Nhe*I restriction site attached (5′-CTGCTAGCCAATCTAAGCTCTCTTC-3′) and 400 bps downstream of the stop codon TAA with a *Sal*I restriction site attached (5′-TGGGTCGACGGTGCTCGATTCGAA-3′). The fragment was fully sequenced before being cloned into pCambia1300 at the *Xba*I and *Sal*I sites. The clone was transferred into GV3101 (pMP90) for plant transformation using the flower-dipping protocol. Independent T1 plants were identified based on hygromycin resistance and analyzed for complementation of the *scord5*-1 and 188G03 (*scord5-2*) mutations.

### Identification of the Tn*5* insertion in the *Pst* DC3118 genome

The site of the Tn*5* insertion in *Pst* DC3118 was identified using iPCR performed according to Huang et al. [43]. Single colonies of *Pst* DC3000 and *Pst* DC3118 were grown overnight in 4 ml of low-salt LB culture media with appropriate antibiotics (*Pst* DC3000: Rif 100 µg/ml; *Pst* DC3118: Rif 100 µg/ml, Km 25 µg/ml). Overnight cultures were centrifuged (5 min at 2000×*g*) and cell pellets were resuspended in 400 µl of TES lysis solution (50 mM Tris-HCl, pH 7.5, 10 mM NaCl, 10 mM EDTA) supplemented with Sarcosyl (final concentration of 1%) and proteinase K (100 µg/ml). Lysates became clear after 45 min, whereupon 225 µl of ice cold NH_4_OAc (7.5 M) was added. Genomic DNA was extracted from lysates and purified using a conventional phenol chloroform extraction protocol [44], resuspended in 10 mM Tris-HCl (pH 8.0) and stored at −20°C. One µg of purified genomic DNA was digested using 20 U of restriction enzyme *Bam*HI (New England Biolabs) for 16 h at 37°C and concentrated by ethanol precipitation before self-ligation using T4 DNA ligase (Promega) at 16°C overnight. The ligation product was used as the template for PCR with primers BR/IR0 and BL/IR0 according to manufacturer's directions (*PfuUltra* II Fusion HS DNA Polymerase, Agilent Technologies) and was isolated by gel electrophoresis (Figure S1). The band was purified using the QIAquick Gel Extraction Kit (Qiagen) and sequenced using the primers IR0 and BL. Amplicon sequencing indicated that the genomic region flanking the Tn*5* insertion corresponds to *cfa6*. We confirmed the Tn*5* insertion locus to be in *cfa6* by PCR amplification of *Pst* DC3118 gDNA using Tn*5*- and *cfa6*-specific primer sets (Figure S1) and amplicon sequencing. The sequences of primers used here are: IR0 (5′-GCCGAAGAGAACACAGATTTAGC-3′), BL (5′-GGGGACCTTGCACAGATAGC-3′), BR (5′-CATTCCTGTAGCGGATGGAGATC-3′), cfa6F (5′-AGTCATGGACGGACAGGTTC-3′), and cfa6R (5′-CCAAGCTCTACGATTCCGAG-3′).

### Callose deposition

Leaves from 4-week-old plants were infiltrated with water or 10 nM flg22 using a needleless syringe. After 24 h, about eight leaves from at least five independent plants were cleared and dehydrated with 100% ethanol. Leaves were fixed in an acetic acid∶ethanol (1∶3) solution for 2 h, sequentially incubated for 15 min in 75% ethanol, 50% ethanol, and 150 mM phosphate buffer, pH 8.0, and then stained overnight at 4°C in 150 mM phosphate buffer (pH 8.0) containing 0.01% (w/v) aniline blue supplemented with carbinicillin (100 µg/ml). After staining, leaves were mounted in 50% glycerol and examined by UV epifluorescence using an Axio Image M1 microscope (Zeiss). Callose quantification was performed using Image J software.

### Oxidative burst in Arabidopsis seedlings

Active oxygen species released by seedlings were assayed by H_2_O_2_-dependent luminescence of luminol [45]. Two-week-old seedlings were transferred from liquid culture into 96-well plates and incubated overnight in 200 µl H_2_O supplemented with carbinicillin (100 µg/ml) at room temperature. The next morning, 100 µl H_2_O containing 20 µM luminol and 1 µg horseradish peroxidase (Sigma) was added and luminescence was measured for 20 min after the addition of the test solutions (100 nM flg22). Luminescence was detected using a Spectra Max L plate reader (Molecular Devices, Sunnyvale, CA). Seedlings were weighed to normalize results.

### Quantification of SA by liquid chromatography-tandem mass spectrometry (LC/MS)

Approximately 100–300 mg (fresh weight) of leaf tissues were frozen in liquid nitrogen, ground, and extracted with 1 ml methanol∶water (1∶1 v/v) containing 0.1% formic acid and 0.1 g L^−1^ butylated hydroxytoluene (BHT) at 4°C for 24 h. Homogenates were mixed and centrifuged at 12,000×*g* for 10 min at 4°C. Supernatants were filtered through 0.2 µm PTFE membrane (Millipore, Bedford, MA) and transferred to autosampler vials. Injections of plant extracts (10 µl per injection) were separated on a fused core Ascentis Express C18 column (2.1×50 mm, 2.7 µm; Supelco, Bellefonte, PA) installed in the column heater of an Acquity Ultra Performance Liquid Chromatography (UPLC) system (Waters Corporation, Milford, MA). A gradient of 0.15% aqueous formic acid (solvent A) and methanol (solvent B) was applied in a 5-min program with a mobile phase flow rate of 0.4 ml/minute. The separation consisted of a linear increase from A∶B (9∶1, v/v) to 100% B. The column, which was maintained at 50°C, was interfaced to a Quattro Premier XE tandem quadrupole mass spectrometer (Waters Corporation, Milford, MA) equipped with electrospray ionization and operated in negative ion mode. The capillary voltage, cone voltage, and extractor voltage were set at 3 kV, 30 V, and 3V, respectively. The flow rates of cone gas and desolvation gas were 100 and 800 L h^−1^, respectively. The source temperature was 120°C and the desolvation temperature was 350°C. Propyl 4-hydroxybenzoate was added as the internal standard for quantification of SA. Transitions from deprotonated molecules to characteristic product ions were monitored for SA (m/z 137>93) and propyl 4-hydroxybenzoate (179>93). Collision energies and source cone potentials were optimized for each transition using QuanOptimize software. Peak areas were integrated, and the analytes were quantified based on standard curves generated from peak area ratios of analytes related to the corresponding internal standard. Data acquisition and processing were performed using Masslynx 4.1 software (Waters, Milford, MA).

### TAIR accession numbers

Sequence data for genes and proteins described in this article can be found in The Arabidopsis Information Resource (TAIR, http://www.arabidopsis.org/) under the following ID numbers: *ACT1* (Arabidopsis actin 1): At2g37620; *OST1*: At4g33950; *SCORD3/EDS5*: At4g39030; *SCORD5/AtGCN20/AtABCF3*: At1g64550.

## Supporting Information

Figure S1 ***Pst*** **DC3118 carries a Tn** ***5*** **insertion in the** ***cfa6*** **gene and does not produce coronatine in planta.** (**A**) A schematic diagram of gene structures surrounding the Tn*5* insertion in the *Pst* DC3118 genome. B, *Bam*HI. (**B**) PCR reactions showing that the Tn*5* insertion is in the genome of *Pst* DC3118 but not that of wild-type *Pst* DC3000. Primers used were specific to *cfa6* and Tn*5*, as indicated in (**A**). (**C**) Detection of coronatine in Col-0 plants infiltrated with *Pst* DC3000 or *Pst* DC3118 bacteria (1×10^8^ CFU/ml) at various time points after infiltration.
(EPS)

Figure S2 **Responses of L** ***er*** **and** ***ost1-2*** **plants to** ***Pst*** **DC3118 (COR-deficient) and** ***Pst*** **DC3000** ***hrcC*** **mutant (T3SS-deficient) when dip-inoculated at 1×10^8^ CFU/ml.** (**A**) Leaf appearance at 3 dpi. (**B**) Bacterial populations at 3 dpi. Statistical analyses for this and following figures are described in MATERIALS and METHODS.
(TIF)

Figure S3 ***scord*** **mutants are not susceptible to** ***Pst*** **DC3000** ***hrcC*** **mutant.** Plants were dip-inoculated at 1×10^8^ CFU/ml and assayed for bacteria populations at 3 dpi. No significant difference was detected between Col-7 and each of the *scord* mutants in Student's t-tests.
(EPS)

Figure S4 **A T-DNA insertion is present in the first intron of** ***EDS5*** **(** ***At4g39030*** **) in the** ***scord3*** **mutant.** (**A**) The exon-intron structure of *EDS5*, showing positions of the T-DNA insertion and *EDS5*- (108 and 109) and T-DNA-specific (T-1 and T-3) primers. (**B**) PCR products generated with indicated primer sets and genomic DNA templates. (**C**) RT-PCR products using *ACT1* (Arabidopsis actin 1; At2g37620)- or *EDS5*-specific primers (108 and 109) and cDNA from Col-7 or *scord3* as template.
(EPS)

Figure S5 **Similar phenotypes of** ***eds5-1*** **and** ***scord3*** **plants.** (**A**) SA and SAG levels in leaves 12 h after infiltration with H_2_O or *Pst* DC3000 at 1×10^8^ CFU/ml. Numbers represent means and standard errors (n = 4). (**B**) Populations of *Pst* DC3118 at day 3 after dip-inoculation at 1×10^8^ CFU/ml. (**C**) and (**D**) Stomata apertures in response to *Pst* DC3118 at 1×10^8^ CFU/ml (**C**) or ABA at 10 µM (**D**).
(EPS)

Figure S6 **Phenotypes of F1 plants from** ***scord3*** **and** ***eds5-1*** **crossings.** (**A**) Eight different F1 plants (designated 1, 2, 3, 5, 6, 7, 8, 9) were selected from five different crosses (designated I, II, III, IV, V) between *scord3* and *eds5-1*. The Col-0 plant was designated as plant 4 in a blind assignment of numbers, and used as a control. (**B**) PCR products amplified with primers T-3 and 109 (see **Figure S4**) and genomic DNA from the nine plants selected in (**A**) as template. (**C**) Stomata apertures in leaf peels of the nine plants selected in (**A**) in response to *Pst* DC3118 at 1×10^8^ CFU/ml. (**D**) SA and SAG levels in the leaves of the nine plants selected in (**A**).
(EPS)

Figure S7 **Stomatal response to elf18 in** ***scord5*** **plants.** Stomatal apertures (µm) were measured after leaf peels had been incubated with buffer or elf18 (100 µM) for 1 h. One hundred µM of elf18 was used to ascertain that elf18-induced stomatal response of *scord5* plants is lost even at this high concentration.
(EPS)

Figure S8 **A phylogenetic tree of the SCORD5 (At1g64550/AtGCN20/AtABCF3) family of proteins.** Sequences were retrieved from The Arabidopsis Information Resource (http://www.arabidopsis.org/), aligned with ClustalX (http://www.clustal.org) and viewed by Dendroscope (http://ab.inf.uni-tuebingen.de/software/dendroscope/).
(EPS)
