# Supplementary figures and images for: A Genetic Screen Reveals Arabidopsis Stomatal and/or Apoplastic Defenses against Pseudomonas syringae pv. tomato DC3000
Source: PLoS Pathog. 2011 Oct 6;7(10):e1002291. doi: 10.1371/journal.ppat.1002291 (PMC3188540; doi:10.1371/journal.ppat.1002291)

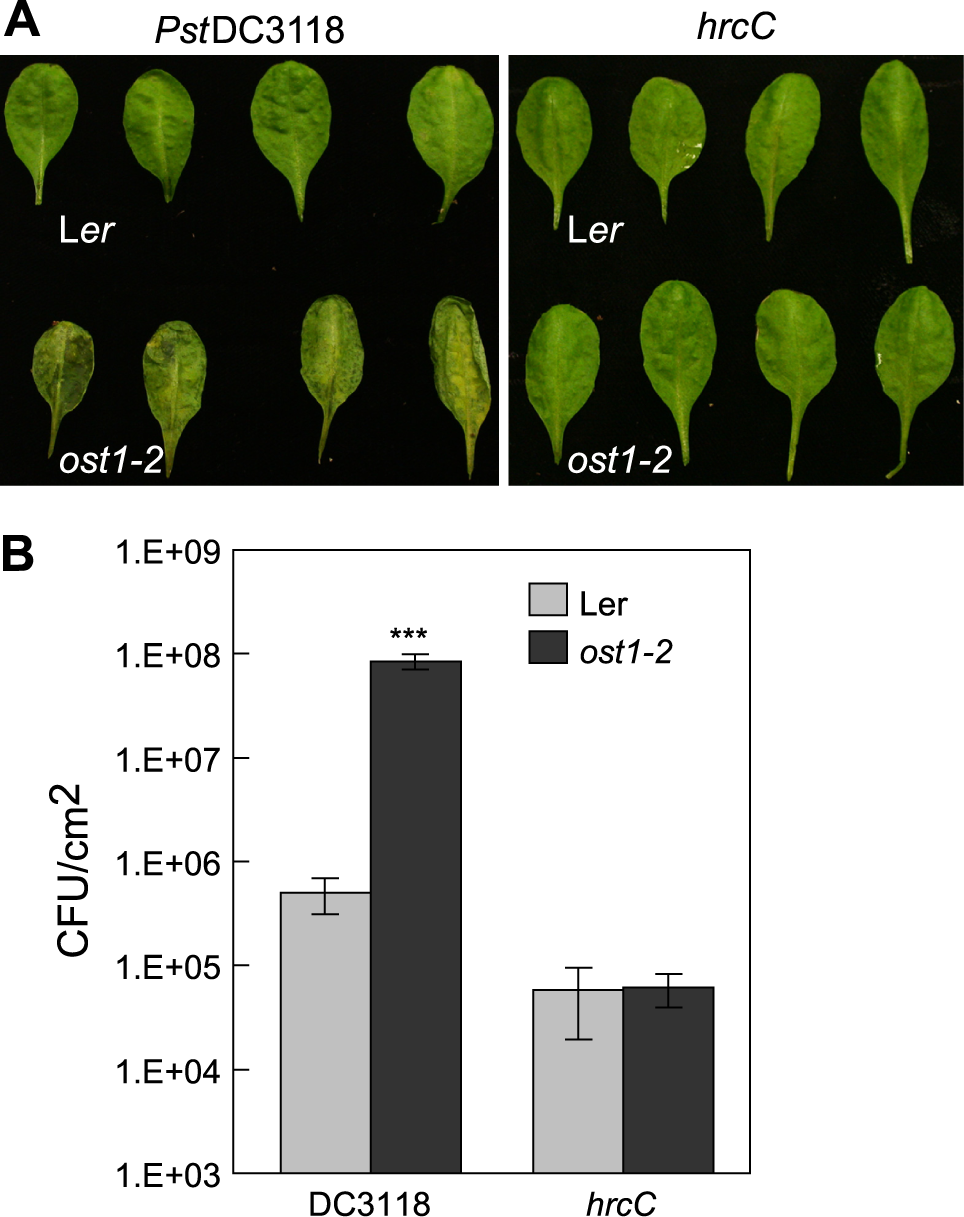

Supplement: Figure S2 — Responses of L er and ost1-2 plants to Pst DC3118 (COR-deficient) and Pst DC3000 hrcC mutant (T3SS-deficient) when dip-inoculated at 1×10^8^ CFU/ml. (A) Leaf appearance at 3 dpi. (B) Bacterial populations at 3 dpi. Statistical analyses for this and following figures are described in MATERIALS and METHODS. (TIF) [file ppat.1002291.s002.tif]
